# Correlation analysis of serum brain-derived neurotrophic factor, matrix metalloproteinase-9, and glutamate levels changes after repetitive transcranial magnetic stimulation intervention with functional improvement in patients with acute ischemic stroke

**DOI:** 10.3389/fneur.2026.1907338

**Published:** 2026-09-17

**Authors:** Xiaohui Li, Yi Sui, Xinru Han, Huimin Guo, Mingyang Wei, Fengjie Liu

**Affiliations:** 1 Department of Neurology, Heji Hospital Affiliated to Changzhi Medical College, Changzhi, China; 2 Department of Intensive Care Unit, The Second Hospital of Dalian Medical University, Dalian, China; 3 Changzhi Medical College, Changzhi, China; 4 Department of General Practice, The Second Hospital of Dalian Medical University, Dalian, China

**Keywords:** acute ischemic stroke, brain-derived neurotrophic factor, glutamate, matrix metalloproteinase-9, motor function, neurological function, repetitive transcranial magnetic stimulation

## Abstract

**Objective:**

To explore the correlation of serum brain-derived neurotrophic factor (BDNF), matrix metalloproteinase-9 (MMP-9), and glutamate (Glu) changes with functional recovery after repetitive transcranial magnetic stimulation (rTMS) in acute ischemic stroke (AIS) patients, so as to provide biological evidence for rTMS efficacy evaluation.

**Methods:**

A retrospective study was conducted on 149 patients with AIS, who were assigned to a control group (*n* = 89, conventional comprehensive treatment) or an rTMS group (*n* = 60, combined rTMS intervention) based on their actual treatment, both for 4 weeks. The two groups were comparable at baseline in age (60.90 ± 2.92 vs. 61.28 ± 3.26 years), gender distribution (54 males vs. 40 males), and admission NIHSS scores [median (IQR): 10.0 (9.0–12.0) vs. 11.0 (9.0–12.0)] (all *P* > 0.05). In the rTMS group, rTMS was initiated within 72 h after stroke onset; the control group did not receive rTMS intervention. Serum levels of BDNF, MMP-9, and Glu were measured before and after treatment in both groups. Neurological and motor functions were assessed using the National Institutes of Health Stroke Scale (NIHSS) and the Fugl-Meyer Assessment (FMA). Pearson correlation was used to analyze the relationships between biomarker changes and functional improvements in the rTMS group, and adverse events were compared between groups.

**Results:**

After 4 weeks, the rTMS group showed significantly greater increase in BDNF and decreases in MMP-9 and Glu than controls (*P* < 0.05), along with lower NIHSS and higher FMA scores (*P* < 0.05). Changes in all three biomarkers significantly correlated with NIHSS reduction and FMA improvement (*P* < 0.001). Adverse events were similar between groups (*P* > 0.05).

**Conclusion:**

: rTMS combined with conventional therapy may be associated with greater improvement in neurological and motor function in AIS, potentially via regulating BDNF, MMP-9, and Glu. These biomarkers may serve as candidate indicators for treatment response. However, given the observational nature of this study, these findings should be interpreted with caution and require confirmation in future randomized controlled trials.

## 1 Introduction

Acute ischemic stroke (AIS) accounts for approximately 70% of all strokes and is characterized by rapid onset and progression, often resulting in debilitating neurological deficits such as hemiplegia, aphasia, and cognitive decline that profoundly impair patients' quality of life and impose substantial burdens on families and society (1–3). Current standard management includes time-windowed intravenous thrombolysis, endovascular recanalization, and pharmacotherapies (e.g., antiplatelet, anticoagulant, and neuroprotective agents), followed by rehabilitation training (4–6). However, neurological recovery remains suboptimal in a subset of patients, underscoring the pressing need for safe and effective adjunctive therapies as well as objective biomarkers for treatment monitoring (7).

Repetitive transcranial magnetic stimulation (rTMS) is a non-invasive neuromodulation technique that uses magnetic fields to modulate cortical neuronal excitability and promote neuroplasticity. It has been widely used in AIS rehabilitation (8, 9). However, its precise mechanisms remain incompletely understood, and are thought to involve modulation of neurotransmitter release, attenuation of inflammatory responses, and upregulation of neurotrophic factors (10–12).

Brain-derived neurotrophic factor (BDNF) is an important neurotrophic factor that promotes neuronal survival, proliferation, and differentiation, enhances neuroplasticity, and plays a key role in neurological repair after ischemic stroke (13). Matrix metalloproteinase-9 (MMP-9) is a type of matrix metalloproteinase that can degrade the basement membrane components of the blood-brain barrier and aggravate cerebral ischemia-reperfusion injury. Its elevated levels correlate with the severity of neurological deficit in AIS (14, 15). Glutamate (Glu), the primary excitatory neurotransmitter in the central nervous system, is massively released and accumulates following ischemic stroke, producing excitotoxicity that leads to neuronal damage and apoptosis, thereby aggravating neurological dysfunction (16, 17).

Although BDNF, MMP-9, and Glu are each critically involved in ischemic stroke pathophysiology, previous studies have largely examined them in isolation, without a systematic evaluation of their combined changes in relation to rTMS efficacy. Therefore, this study was designed to investigate the changes in serum BDNF, MMP-9, and Glu levels in AIS patients receiving rTMS, and to explore their correlations with concurrent improvements in neurological and motor function, thereby providing potential biological evidence for rTMS efficacy assessment.

## 2 Materials and methods

### 2.1 Study subjects

A total of 220 patients with AIS admitted to the Department of Neurology of our hospital from January 2025 to January 2026 were retrospectively screened. After applying the inclusion and exclusion criteria, 149 patients were enrolled as study subjects. Based on the actual treatment regimens they received, the patients were divided into a control group (*n* = 89, receiving conventional comprehensive therapy) and an rTMS group (*n* = 60, receiving conventional therapy combined with rTMS intervention). This study was approved by the Heji Hospital Affiliated to Changzhi Medical College Ethics Committee of our hospital and complied with the Declaration of Helsinki. Inclusion criteria: meeting the diagnostic criteria for AIS as defined in the Chinese Guidelines for the Diagnosis and Treatment of Acute Ischemic Stroke (2023 Edition), and confirmed by cranial CT or MRI; first-ever ischemic stroke; time from onset ≤ 72 h; complete clinical data; patients were conscious and able to cooperate with treatment and examinations. Exclusion criteria: presence of severe cardiac, hepatic, renal, pulmonary, or other organ dysfunction; presence of other neurological diseases such as cerebral hemorrhage, brain tumor, or epilepsy; contraindications to rTMS treatment (e.g., presence of a cardiac pacemaker, metal implants, *etc*.); presence of psychiatric disorders or cognitive impairment that prevented cooperation; concomitant craniocerebral injury or other central nervous system diseases; pregnant or lactating women; receipt of other neuromodulation therapies within the previous 3 months.

### 2.2 Treatment methods

Both groups received conventional comprehensive treatment, which specifically included: Antiplatelet therapy: oral administration of aspirin enteric-coated tablets (100 mg/time, once daily) or clopidogrel tablets (75 mg/time, once daily); Improvement of cerebral circulation: intravenous infusion of butylphthalide and sodium chloride injection (100 mL/time, twice daily) or edaravone injection (30 mg/time, twice daily); Neurotrophic support: oral administration of mecobalamin tablets (0.5 mg/time, three times daily) and vitamin B1 tablets (10 mg/time, three times daily); Control of risk factors such as blood pressure, blood glucose, and blood lipids, along with symptomatic supportive treatment.

The rTMS group received additional rTMS intervention on the basis of conventional treatment. A Rapid2 rTMS therapeutic apparatus (Magstim, UK) with a figure–eight coil was used. The stimulation frequency was 10 Hz, and the stimulation intensity was 80% of the motor threshold, which was determined using a standard procedure. The stimulation site was the primary motor cortex (M1 area) of the affected hemisphere. Each session lasted 20 min (10 s of stimulation followed by 5 s of rest), once daily, 5 days per week with 2 days of rest, for a total of 4 weeks. The control group received only conventional comprehensive treatment without rTMS stimulation, with the same treatment duration as the rTMS group.

### 2.3 General data collection

The general information of the patients, such as gender, age, body mass index (BMI), smoking history, drinking history, hypertension history, diabetes history, coronary heart disease history, and affected hemisphere, was reviewed and extracted through the electronic medical record system. Initial clinical manifestations at admission, including hemiplegia, aphasia/dysarthria, limb sensory disturbance, dizziness/vertigo, and ataxia, were also collected.

#### 2.3.1 NIHSS scale assessment

The National Institutes of Health Stroke Scale (NIHSS) was used to assess the degree of neurological deficits. This scale includes 11 dimensions such as consciousness, gaze, limb movement, sensation, and speech, with a total score ranging from 0 to 42. A higher score indicates more severe neurological impairment. All assessments were independently conducted by two trained attending neurologists blinded to group allocation. The final score for each patient was the mean of the two raters' scores, with any discrepancies adjudicated by a senior neurologist (18).

#### 2.3.2 FMA motor function score assessment

The Fugl-Meyer Assessment (FMA) scale was used to evaluate patients before and after intervention. The scale includes 33 items for the upper limbs and 17 items for the lower limbs. Each item is scored from 0 to 2, with a maximum total score of 100. A higher score indicates better limb motor function. Similarly, FMA assessments were performed by the same two independent trained attending neurologists, who were blinded to the patients' group allocation, and the mean score of the two raters was used for analysis (19).

### 2.4 Enzyme-linked immunosorbent assay (ELISA)

Peripheral venous blood (5 mL) was collected from patients in the fasting state in the early morning before treatment and after 4 weeks of treatment. After standing at room temperature for 30 min, the blood samples were centrifuged at 3,000 r/min for 10 min. The serum was separated and stored in a refrigerator at −80 °C for later use. All samples were tested in the same batch to avoid repeated freezing and thawing. Serum levels of BDNF, MMP-9, and Glu were measured according to the instructions of ELISA kits. The human brain-derived neurotrophic factor (BDNF) ELISA kit (Catalog No.: CSB-E04501h, detection range: 0.313–20 ng/mL) was purchased from Wuhan Yansheng Biotechnology Co., Ltd. The human matrix metalloproteinase-9 (MMP-9) ELISA kit (Catalog No.: QT-EH0238, detection range: 0.313–20 ng/mL) was purchased from Wuhan Fine Biotechnology Co., Ltd. Serum glutamate (Glu) levels were detected by high-performance liquid chromatography (HPLC), and the kit was purchased from Nanjing Jiancheng Bioengineering Institute. All procedures were strictly performed according to the kit instructions.

### 2.5 Statistical analysis

SPSS 27.0 statistical software (SPSS, Inc., Chicago, IL, USA) and GraphPad Prism 9.5.0 software (GraphPad Software Inc., San Diego, CA, USA) were used for statistical analysis and graphing. The Shapiro–Wilk test was used to test for normal distribution. Measurement data conforming to a normal distribution were expressed as mean ± standard deviation. Independent samples *t*-test was used for comparison between two groups, and paired *t*-test was used for comparison before and after treatment. Measurement data not conforming to a normal distribution were expressed as median (interquartile range). The Mann–Whitney *U*-test was used for comparison between groups, and the Wilcoxon signed-rank test was used for comparison before and after treatment within the same group. Count data were expressed as numbers and percentages, and the chi-square test was used for comparison between groups. All *P* values were two-tailed, and *P* < 0.05 was considered statistically significant.

## 3 Results

### 3.1 Comparison of general data between the two groups

A total of 149 patients with AIS were enrolled in this study. According to the actual treatment regimens they received, the patients were divided into a control group (*n* = 89, receiving conventional comprehensive therapy) and an rTMS group (*n* = 60, receiving conventional therapy combined with rTMS intervention). Comparison of general data between the two groups showed (Table 1) that there were no statistically significant differences in age, gender BMI, smoking history, drinking history, history of hypertension, history of diabetes mellitus, history of coronary heart disease, or affected hemisphere between the two groups (*P* > 0.05). Furthermore, the distributions of initial clinical manifestations at admission, including hemiplegia, aphasia/dysarthria, limb sensory disturbance, dizziness/vertigo, and ataxia, were also comparable between the two groups (all *P* > 0.05, Table 1), indicating comparability.

**Table 1:** Comparison of general data between the two groups.

Variable	Control group (*n* = 89)	rTMS group (*n* = 60)	t/χ^2^	*P*
Age (years)	60.90 ± 2.92	61.28 ± 3.258	0.753	0.453
Gender (*n*, %)				
Male	54 (60.67%)	40 (66.67%)	0.553	0.457
Female	35 (39.33%)	20 (33.33%)		
BMI (kg/m^2^)	24.83 ± 1.17	25.02 ± 1.24	0.969	0.334
Smoking history (*n*, %)	36 (40.45%)	27 (45.00%)	0.304	0.581
Drinking history (*n*, %)	28 (31.46%)	21 (35.00%)	0.203	0.652
Hypertension history (*n*, %)	50 (56.18%)	38 (63.33%)	0.759	0.384
Diabetes mellitus history (*n*, %)	19 (21.35%)	15 (25.00%)	0.271	0.602
Coronary heart disease history (*n*, %)	14 (15.73%)	13 (21.67%)	0.851	0.356
Affected hemisphere (*n*, %)				
Left	38 (42.70%)	28 (46.67%)	0.229	0.632
Right	51 (57.30%)	32 (53.33%)		
Initial clinical manifestations at admission (*n*, %)				
Hemiplegia	76 (85.39%)	53 (88.33%)	0.267	0.606
Aphasia/dysarthria	31 (34.83%)	24 (40.00%)	0.411	0.521
Limb sensory disturbance	26 (29.21%)	19 (31.67%)	0.102	0.749
Dizziness/vertigo	18 (20.22%)	14 (23.33%)	0.205	0.650
Ataxia	9 (10.11%)	7 (11.67%)	0.090	0.764

BMI, body mass index.

The Shapiro-Wilk test was used for normality testing. Measurement data conforming to normal distribution were expressed as mean ± standard deviation, and comparison between two groups was performed using the independent samples *t*-test. Count data were expressed as numbers and percentages, and comparison between groups was performed using the chi-square test.

*P* < 0.05 was considered statistically significant.

### 3.2 Comparison of serum BDNF, MMP-9, and Glu levels between the two groups

Before intervention, there were no statistically significant differences in serum BDNF, MMP-9, or Glu levels between the two groups (*P* > 0.05). After 4 weeks of intervention, serum BDNF levels significantly increased, while serum MMP-9 and Glu levels significantly decreased in both groups compared with before intervention, with statistically significant differences (all *P* < 0.001). Moreover, the serum BDNF level in the rTMS group was significantly higher than that in the control group, and the serum MMP-9 and Glu levels in the rTMS group were significantly lower than those in the control group, with statistically significant differences (all *P* < 0.05); (Figure 1).

**Figure 1:**
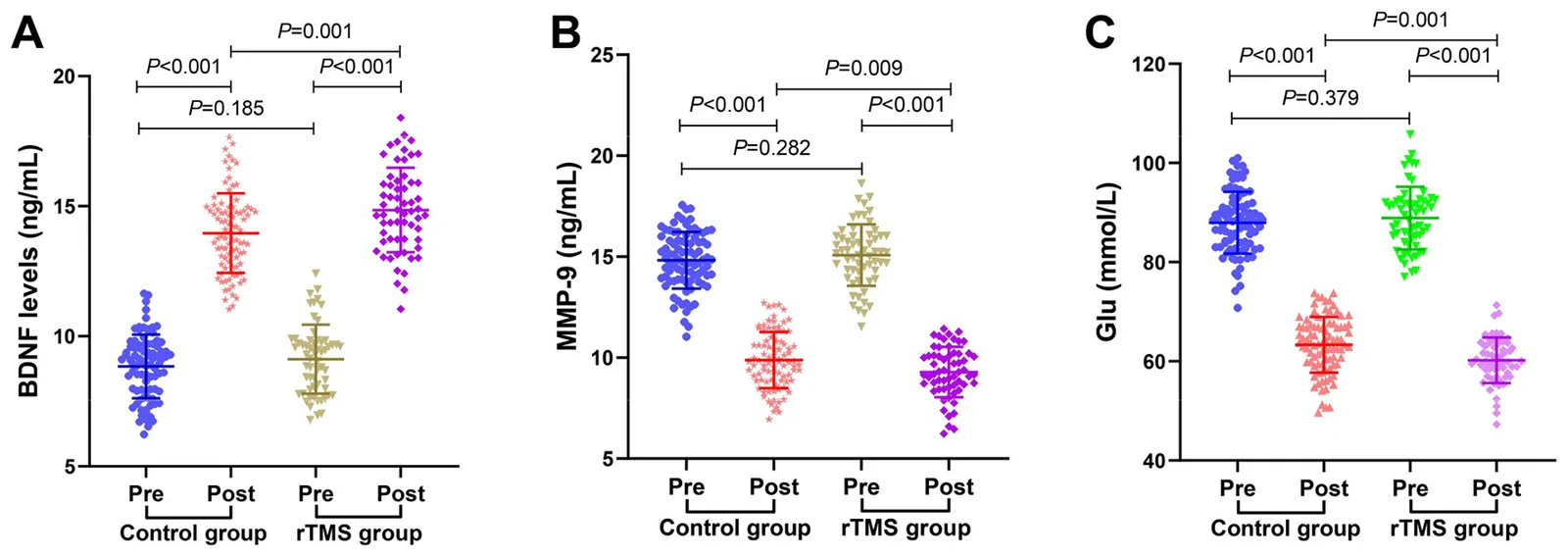
Comparison of serum BDNF, MMP-9, and Glu levels between the two groups. BDNF, brain-derived neurotrophic factor; MMP-9, matrix metalloproteinase-9; Glu, glutamate. The Shapiro-Wilk test was used for normality testing. Measurement data conforming to normal distribution in panels **(A–C)** are expressed as mean ± standard deviation. Paired *t*-test was used for comparisons before and after treatment, and independent samples *t*-test was used for between-group comparisons. *P* < 0.05 was considered statistically significant.

### 3.3 Comparison of NIHSS scores between the two groups

Before intervention, there was no statistically significant difference in NIHSS scores between the two groups (*P* > 0.05). After 4 weeks of intervention, NIHSS scores in both groups decreased significantly compared with before intervention, with statistically significant differences (all *P* < 0.001). Moreover, the NIHSS score in the rTMS group was significantly lower than that in the control group (*P* < 0.05); (Figure 2). This indicates that rTMS combined therapy can more significantly improve the degree of neurological impairment in patients with AIS.

**Figure 2:**
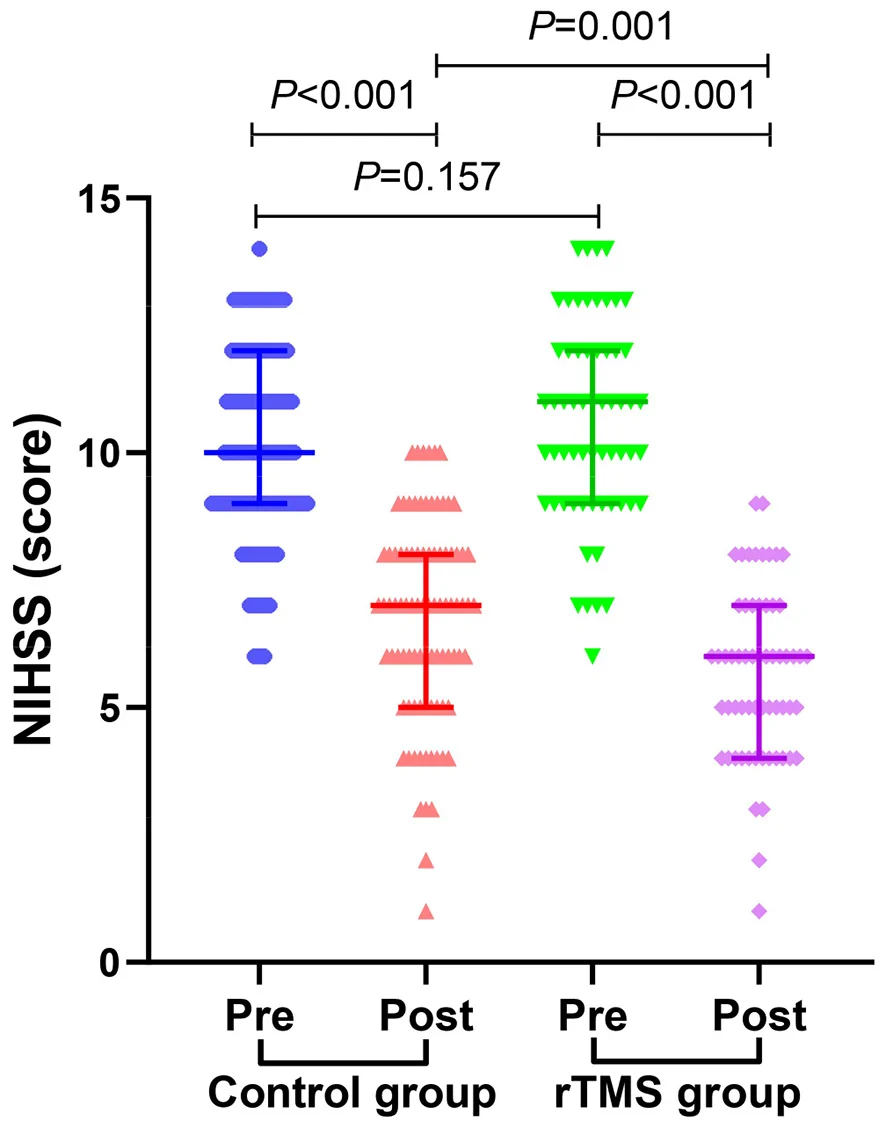
Comparison of NIHSS scores between the two groups. NIHSS, national institutes of health stroke scale. The Shapiro-Wilk test was used for normality testing. The data in the figure did not conform to a normal distribution and are expressed as median (interquartile range). The Mann-Whitney *U*-test was used for between-group comparisons, and the Wilcoxon signed-rank test was used for within-group comparisons before and after treatment. *P* < 0.05 was considered statistically significant.

### 3.4 Comparison of FMA motor function scores between the two groups

Before intervention, there were no statistically significant differences in total FMA scores or subscores for upper and lower limbs between the two groups (all *P* > 0.05). After 4 weeks of intervention, total FMA scores and subscores for upper and lower limbs in both groups increased significantly compared with before intervention, with statistically significant differences (all *P* < 0.001). Moreover, FMA motor function scores in the rTMS group were significantly higher than those in the control group (all *P* < 0.05); (Figure 3). This suggests that rTMS combined with conventional therapy can more effectively improve patients' limb motor function and promote the recovery of motor ability.

**Figure 3:**
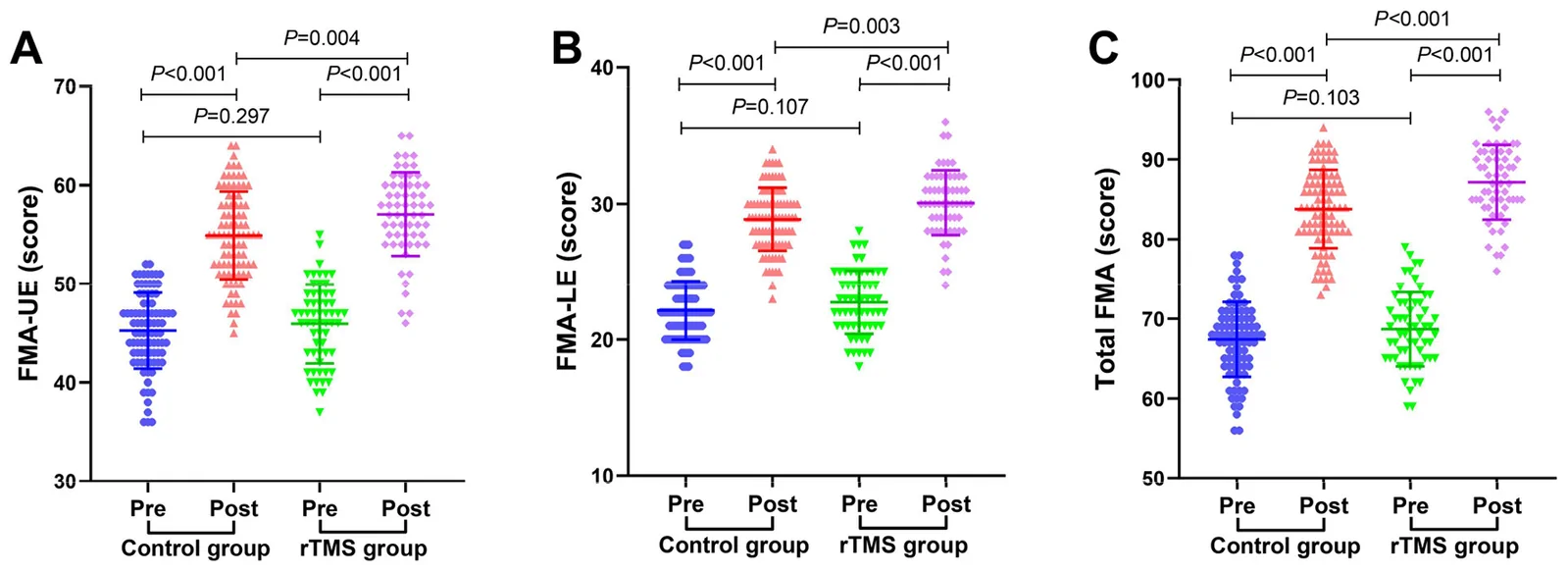
Comparison of FMA motor function scores between the two groups **(A)** FMA-UE, Fugl-Meyer Assessment of the Upper Extremity; **(B)** FMA-LE, Fugl-Meyer Assessment of the Lower Extremity; **(C)** FMA, Fugl-Meyer Assessment. The Shapiro-Wilk test was used for normality testing. The data in the figure did conform to a normal distribution and are expressed as mean ± standard deviation. Paired *t*-test was used for comparisons before and after treatment, and independent samples *t*-test was used for between-group comparisons. *P* < 0.05 was considered statistically significant.

### 3.5 Correlation analysis of changes in BDNF, MMP-9, and Glu with NIHSS improvement

To explore the relationship between the changes of serum biomarkers and the improvement of neurological function, the correlations between the changes of BDNF, MMP-9, Glu and the improvement of NIHSS were analyzed in the rTMS treatment group of patients. Pearson correlation analysis performed on the rTMS group showed (Figure 4) that the magnitude of BDNF increase (ΔBDNF) was significantly negatively correlated with the magnitude of NIHSS score reduction (ΔNIHSS); (*r* = −0.628, *P* < 0.001); the magnitude of MMP-9 reduction (ΔMMP-9) was significantly positively correlated with ΔNIHSS (*r* = 0.659, *P* < 0.001); and the magnitude of Glu reduction (ΔGlu) was also significantly positively correlated with ΔNIHSS (*r* = 0.524, *P* < 0.001). These results indicate that changes in serum BDNF, MMP-9, and Glu levels are closely associated with neurological improvement and may serve as potential biological indicators for evaluating the efficacy of rTMS.

**Figure 4:**
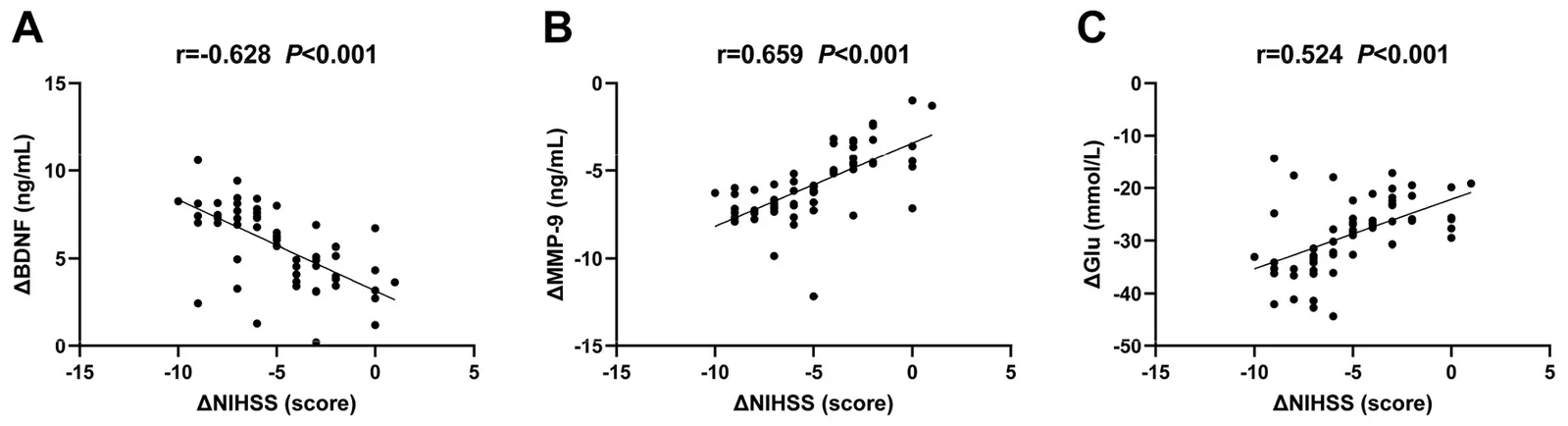
Scatter plots of changes in BDNF, MMP-9, and Glu with NIHSS improvement. **(A)** Pearson correlation between ΔBDNF and ΔNIHSS; **(B)** Pearson correlation between ΔMMP-9 and ΔNIHSS; **(C)** Pearson correlation between ΔGlu and ΔNIHSS. ΔBDNF: magnitude of BDNF increase (post-treatment minus pre-treatment); ΔMMP-9: magnitude of MMP-9 reduction (post-treatment minus pre-treatment); ΔGlu: magnitude of Glu reduction (post-treatment minus pre-treatment); ΔNIHSS: magnitude of NIHSS score reduction (post-treatment minus pre-treatment). *P* < 0.05 was considered statistically significant.

### 3.6 Correlation analysis of changes in BDNF, MMP-9, and Glu with FMA improvement

Pearson correlation analysis performed on the rTMS treatment group showed (Figure 5) that ΔBDNF was significantly positively correlated with the magnitude of FMA score increase (ΔFMA); (*r* = 0.588, *P* < 0.001); ΔMMP-9 was significantly negatively correlated with ΔFMA (*r* = −0.625, *P* < 0.001); and ΔGlu was also significantly negatively correlated with ΔFMA (*r* = −0.505, *P* < 0.001). This further confirms that changes in the above three serum molecules are highly correlated with the degree of motor function recovery, supporting their use as important reference indicators for evaluating the response to rTMS treatment.

**Figure 5:**
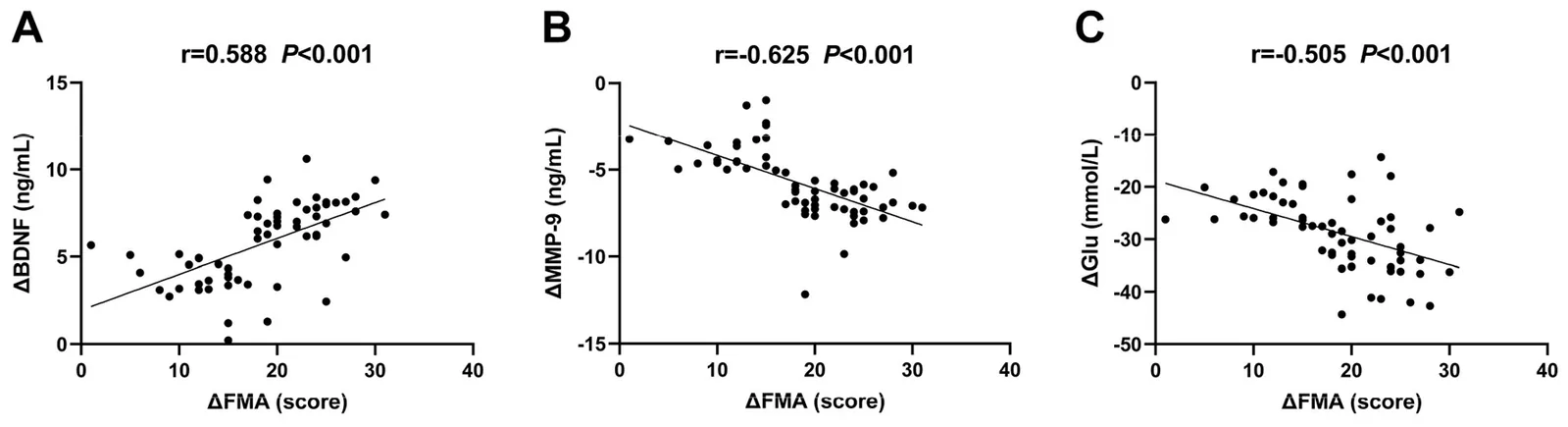
Scatter plots of changes in BDNF, MMP-9, and Glu with FMA improvement. **(A)** Pearson correlation between ΔBDNF and ΔFMA; **(B)** Pearson correlation between ΔMMP-9 and ΔFMA; **(C)** Pearson correlation between ΔGlu and ΔFMA. ΔBDNF: magnitude of BDNF increase (post-treatment minus pre-treatment); ΔMMP-9: magnitude of MMP-9 reduction (post-treatment minus pre-treatment); ΔGlu: magnitude of Glu reduction (post-treatment minus pre-treatment); ΔFMA: magnitude of FMA score increase (post-treatment minus pre-treatment). *P* < 0.05 was considered statistically significant.

### 3.7 Comparison of adverse reactions between the two groups

During the treatment period, three patients in the rTMS group experienced mild headache and one patient experienced transient dizziness, all of which resolved spontaneously after rest without affecting subsequent treatment. No seizures or other serious adverse events occurred. In the control group, two patient experienced mild headache, with no other significant adverse reactions. The incidence of adverse reactions was 2.25% in the control group and 6.67% in the rTMS group, with no statistically significant difference between the two groups (χ^2^ = 1.811, *P* = 0.178); (Table 2). This indicates that rTMS combined with conventional therapy has a favorable safety profile, with mild and manageable adverse reactions.

**Table 2:** Comparison of adverse reactions between the two groups.

Adverse reaction	Control group (*n* = 89)	rTMS group (*n* = 60)	χ^2^	*P*
Mild headache (*n*, %)	2 (2.25%)	3 (5.00%)		
Transient dizziness (*n*, %)	0 (0.00%)	1 (1.67%)		
Total (*n*, %)	2 (2.25%)	4 (6.67%)	1.811	0.178

Count data are expressed as numbers and percentages. The chi-square test was used for between-group comparisons.

*P* < 0.05 was considered statistically significant.

## 4 Discussion

The pathogenesis of AIS is complex, primarily involving thrombosis or atherosclerosis of cerebral arteries caused by multiple factors, leading to luminal stenosis or even occlusion, which results in cerebral ischemia and hypoxia, subsequently causing neurological deficits. These deficits manifest as motor, swallowing, and speech dysfunctions, severely affecting patients' quality of life and imposing heavy burdens on families (20, 21). As a non-invasive neuromodulation technique, rTMS has shown promising application prospects in the rehabilitation of AIS (22). In this study, we systematically observed changes in serum BDNF, MMP-9, and Glu levels before and after 4 weeks of rTMS intervention in patients with AIS, simultaneously evaluated improvements in neurological function (NIHSS) and motor function (FMA), and further explored the correlations between the magnitudes of change in these serum biomarkers and functional improvements.

Our results showed that after intervention, the rTMS group exhibited a significantly greater reduction in NIHSS scores and a significantly greater increase in FMA motor function scores compared with the control group receiving conventional treatment alone, confirming that rTMS can effectively promote neurological and motor function rehabilitation in patients with AIS. These clinical benefits are consistent with findings from multiple systematic reviews and meta-analyses (23, 24). From the perspective of neuromodulation mechanisms, this study applied 10 Hz high-frequency rTMS to the M1 area of the affected hemisphere, which can induce long-term potentiation (LTP)-like plastic changes in the cortex, significantly enhancing the connectivity strength of cerebral neural networks and contributing to the recovery of upper limb motor function in stroke patients (25, 26). Within the theoretical framework of the interhemispheric competition model, increasing the excitability of the affected hemisphere helps correct the transcallosal inhibition state in which the unaffected hemisphere overly inhibits the affected hemisphere after AIS, gradually restoring coordination between the bilateral cortical functional networks (27). Previous studies have confirmed that the degree of decreased cortical excitability in the affected M1 area after ischemic stroke is closely correlated with the severity of limb motor deficits. Therefore, targeting the affected M1 area with high-frequency magnetic stimulation can specifically correct local excitability deficits, representing an important pathway for improving motor function (28, 29). This study found that rTMS intervention significantly upregulated the expression level of serum BDNF in peripheral blood. BDNF is a key neurotrophic factor regulating neuronal survival, differentiation, and synaptic remodeling. After ischemic stroke, the ischemic microenvironment inhibits BDNF gene transcription and protein synthesis, hindering the self-repair of damaged brain tissue (30, 31). This study confirms that high-frequency rTMS can activate the BDNF/TrkB signaling pathway through neuroelectrophysiological regulation, upregulate the synthesis and release of endogenous BDNF, promote synaptic structural remodeling via downstream signaling pathways, induce LTP formation, and improve neuroplasticity in the ischemic area (32, 33). This represents an important molecular basis for rTMS-assisted neurological repair and is mutually corroborated by previous basic animal experiments and small-sample clinical studies (34, 35).

After rTMS intervention, serum MMP-9 levels in the combination group significantly decreased, with a greater magnitude of reduction than that in the control group. As a key member of the matrix metalloproteinase family, MMP-9 is a core mediator of blood-brain barrier (BBB) injury. Cerebral ischemia-reperfusion injury can activate the NF-κB signaling pathway, inducing microglia and vascular endothelial cells to secrete large amounts of MMP-9, resulting in markedly increased BBB permeability, extensive exudation of vasogenic edema fluid and inflammatory cytokines, further exacerbating the inflammatory cascade injury around the infarct core, and significantly increasing the risk of hemorrhagic transformation (36, 37). Clinical studies have also confirmed that serum MMP-9 levels in patients with acute ischemic stroke are independently correlated with early neurological deterioration, infarct volume expansion, and poor prognosis (38). It has been reported that high-frequency rTMS can reduce active MMP9/pro-MMP9 in the injured spinal cord, thereby alleviating blood-spinal cord barrier disruption, inhibiting local inflammatory infiltration, and improving motor function of affected limbs (39). Therefore, it is hypothesized that rTMS may exert protective effects against secondary brain tissue injury by inhibiting the abnormal expression of MMP-9, protecting the BBB, and suppressing the local inflammatory cascade reaction.

Furthermore, Glu is the primary excitatory neurotransmitter in the central nervous system. After ischemic stroke, cellular energy metabolism disorders lead to massive extracellular accumulation of glutamate, which overactivates excitatory receptors such as NMDA and AMPA, inducing calcium overload-mediated excitotoxic neuronal apoptosis. This excitotoxicity is an important contributor to progressive neurological injury in the later stage of ischemic stroke (40). In this study, the rTMS group showed a more pronounced decrease in serum Glu levels after treatment, indicating that rTMS can regulate the central glutamate-γ-aminobutyric acid (GABA) neurotransmitter balance, reduce excessive glutamate release, and alleviate excitotoxic injury (41, 42). At the same time, rTMS, relying on NMDA receptor-dependent plasticity pathways, optimizes synaptic transmission efficacy, improves neural circuit function on the basis of reasonably regulating glutamatergic neurotransmission, and achieves dual effects of neuroprotection and plastic remodeling (41).

Correlation analysis showed that the magnitude of BDNF increase in the rTMS group was significantly correlated with NIHSS reduction and FMA increase, and the magnitudes of MMP-9 and glutamate reduction were also highly correlated with improvements in neurological and motor functions, suggesting that all three biomarkers can serve as potential objective biological markers for evaluating the efficacy of rTMS. Current clinical efficacy assessments mostly rely on functional scales such as NIHSS and FMA, which are susceptible to subjective factors such as rater experience and patient cooperation. Previous studies have mostly used BDNF, MMP-9, or Glu as single biomarkers for assessing the condition or prognosis of ischemic stroke. However, this study is the first to combine all three for evaluating rTMS efficacy. The results indicate that combined detection is more stable and comprehensive than single indicators, can objectively quantify *in vivo* pathophysiological changes and the degree of treatment response, and provides a reliable objective reference for formulating individualized rehabilitation plans and dynamically monitoring efficacy in clinical practice.

Regarding safety, adverse reactions in the rTMS group in this study were mainly mild headache and transient dizziness, with an incidence of 6.67%, showing no statistically significant difference from the control group. The symptoms were mild and resolved spontaneously, with no adverse events such as epilepsy or severe neural stimulation. This finding is consistent with the conclusion that rTMS has good safety and tolerability in stroke rehabilitation, further confirming that rTMS with standardized stimulation parameters is safe and feasible for patients with AIS and can be used as a routine clinical adjunctive rehabilitation measure.

This study has certain limitations. First, this is a single-center study with a limited sample size, and the conclusions still need to be further validated by multi-center, large-sample, double-blind randomized controlled trials. Second, this study only observed short-term efficacy over 4 weeks and lacked medium- to long-term follow-up of 3 months or more, making it impossible to determine the association between changes in the above serum biomarkers and patients' long-term functional prognosis and quality of life. Third, we did not perform subgroup analyses based on stroke subtypes, such as thalamic infarction, large vessel occlusion, or lacunar infarction. Given that acute ischemic stroke encompasses heterogeneous pathological entities with different locations and mechanisms of injury, the patterns of neurological impairment and biomarker responses to rTMS may vary across subtypes. However, due to the retrospective nature and limited sample size of this study, we were unable to conduct reliable subgroup analyses. Fourth, we acknowledge that we did not perform subgroup analyses or multivariable regression models to evaluate whether baseline patient characteristics—such as age, sex, BMI, affected hemisphere, or other clinical variables—influenced treatment response to rTMS, nor did we conduct further adjusted analyses incorporating additional potential confounders (e.g., cardiovascular risk factors and rehabilitation intensity). Although the baseline characteristics were well balanced between the two groups (all *P* > 0.05), which helped to control for potential confounding to some extent, the relatively small sample size (*n* = 60 in the rTMS group) and the retrospective nature of this study precluded reliable stratification or adjustment for multiple covariates without risking overfitting. Therefore, our results should be interpreted as exploratory. Future prospective studies with larger cohorts and more comprehensive adjusted analyses are warranted to identify independent predictors of rTMS efficacy and to determine which patient subgroups are most likely to benefit from this intervention, thereby enhancing the robustness and clinical applicability of the findings. In addition, the effects of different rTMS parameters, infarct locations, etiologies, and baseline functional levels on efficacy were not explored, and potential confounding factors may exist. Future studies could expand the sample size, extend the follow-up period, optimize rTMS treatment parameters, and incorporate multimodal assessment tools such as neuroimaging and electrophysiology to establish a more complete evaluation system for rTMS efficacy in acute ischemic stroke, promoting its more precise and standardized application in clinical rehabilitation.

## 5 Conclusion

This study not only validates the clinical value of rTMS in improving neurological and motor functions in AIS patients, more importantly, but also elucidates its potential molecular mechanisms and identifies a potential biomarker panel (BDNF, MMP-9, and Glu) for treatment monitoring. These findings lay a solid theoretical and practical foundation for deepening the understanding of the neuromodulatory mechanisms of rTMS, optimizing comprehensive treatment strategies for AIS, and developing objective efficacy evaluation systems.

## Data Availability

The original contributions presented in the study are included in the article/Supplementary material, further inquiries can be directed to the corresponding author.
